# Association between psychosocial distress with cardio metabolic risk factors and liver enzymes in a nationally-representative sample of Iranian children and adolescents: the CASPIAN-III study

**DOI:** 10.1186/2251-6581-13-44

**Published:** 2014-03-06

**Authors:** Mostafa Qorbani, Roya Kelishadi, Ehsaneh Taheri, Mohammad Esmaeil Motlagh, Seyed Masoud Arzaghi, Gelayol Ardalan, Mohammad Chinian, Minoosadat Mahmoudarabi, Aziz Rezapoor, Hamid Asayesh, Bagher Larijani, Mohammad Reza Amini, Ramin Heshmat

**Affiliations:** 1 Department of Public Health, Alborz University of Medical Sciences, Karaj, Iran; 2 Department of Epidemiology, Iran University of Medical Sciences, Tehran, Iran; 3 Pediatrics Department, Child Growth and Development Research Center, and Faculty of Medicine, Isfahan University of Medical Sciences, Isfahan, Iran; 4 Endocrinology and Metabolism Research Center, Endocrinology and Metabolism Clinical Sciences Institute, Tehran University of Medical Sciences, Tehran, Iran; 5 Pediatrics Department, Ahvaz Jundishapur University of Medical Sciences, Ahvaz, Iran; 6 Elderly Health Research Center, Endocrinology and Metabolism Population Sciences Institute, Tehran University of Medical Sciences, Tehran, Iran; 7 Department of Youths, Adolescents & School Health, Bureau of Population, Family and School Health, Ministry of Health and Medical Education, Tehran, Iran; 8 Bureau of Health and Fitness, Ministry of Education and Training, Tehran, Iran; 9 Hospital Management Research Center, Iran University of Medical Science, Tehran, Iran; 10 Department of Medical Emergencies, Qom University of Medical Sciences, Qom, Iran; 11 Epidemiology Department, Chronic Diseases Research Center, Endocrinology and Metabolism Population Sciences Institute, Endocrinology and Metabolism Clinical Sciences Institute, Tehran University of Medical Sciences, Tehran, Iran

**Keywords:** Psychosocial distress, Cardio metabolic risk factor, Adolescents

## Abstract

**Background:**

The present study was designed to evaluate association of psychosocial distress with cardio metabolic risk factors and liver enzymes in Iranian children and adolescents.

**Method:**

This nationwide study was conducted as the third survey of the school-based surveillance system that was conducted among 5593 school students, 10–18 years in Iran. High triglyceride (TG), high fasting blood sugar (FBS), high total cholesterol (TC), high low-density lipoprotein cholesterol (LDL-C), low high-density lipoprotein cholesterol (HDL-C), hypertension (HTN), generalized obesity and abdominal obesity were considered as cardio metabolic risk factors and alanine transaminase (ALT) and aspartate aminotransferase (AST) were considered as liver enzymes. Data were analyzed using multiple logistic regression (MLR) analysis.

**Result:**

Psychosocial distress was detected in2027 (71.2%) of boys and 1759 (63.3%) of girls. Among boys, the mean of LDL, AST and DBP were higher and the mean FBS and HDL were lowering those with psychiatric distress than their other counterparts. Girls with psychosocial distress had significantly higher mean of HDL and FBS than those without psychiatric distress. Psychosocial distress significantly increased the odds of high LDL (OR = 2.36, 95%CI 1.53, 3.64), high FBS (OR = 1.23, 95%CI 1.02, 1.49) and low HDL (OR = 1.65, 95%CI 1.41, 1.95).

**Conclusion:**

Psychosocial distress in adolescents is associated with increased risk of some cardio metabolic risk factors.

## Introduction

In addition to socioeconomic status, genetic factors and individual lifestyles, psychosocial parameters have significant impact on health [1]. Psychosocial distressare one of the most common health problems in all over the world [2]. According to the World Health Organization (WHO) reports, one in four people develop some kind of mental illness at some point in their lives [3].

Several studies have been conducted to evaluate mental health status in Iran. Results of Emamiet al. study on in 4599 adolescent mental health status shown that considerable proportion of adolescent students experience mental distress with higher rates in girls compared to boys [4]. First and only systematic review among high-school students in Iran concluded that prevalence rates of any mental distress were reported in a wide range from 4.34% to16.6% in studies using diagnostic instruments to 34.4% in studies using screening instruments [5].

People with severe mental distress have a 2–3 fold increased risk of dying from cardiovascular disease (CVD) [6]. In patients with psychotic problem the rates of medical co-morbidity and premature mortality are higher compared to general populations. This population has a higher prevalence of CVD, metabolic syndrome (MetS) and CVD risk factors such as diabetes, hypertension, dyslipidemia, obesity and smoking compare to general populations. Therefore this population has a reduced life expectancy and higher rate of morbidity and mortality compared to general populations. Psychosocial variables are nontraditional risk factors of CHD (coronary heart disease) that have often been ignored.

Because of early age of onset and the chronic course of psychosocial distress, the prevention, detection, and treatment of these diseases in childhood and adolescent age is very important [7]. Limited evidence exists about the association between psychosocial distress with cardio metabolic risk factors in children and adolescents. Nationwide studies revealed high prevalence of cardio metabolic risk factors and elevated liver enzymes in Iranian children and adolescents [8-11]. In this study we investigated the association of psychosocial distress with cardio metabolic risk factors and liver enzymes among 10-18- year-old adolescents in a nationwide study in Iran.

## Methods

The data used in this study were obtained as a part of the third national Iranian survey (2009–2010), Childhood and Adolescence Surveillance and Prevention of Adult Non-communicable disease (CASPIAN).

The methodology details including data collection process and sampling frame have been reported previously [12] and here we present the methods in brief. The present study was performed among 5593 school students (2,812 boys and 2,781 girls) aged 10–18 years, living in urban and rural areas in 27 provinces of Iran who were selected via multistage-random cluster sampling method. Eligible schools in our study were stratified according to the information bank of the Ministry of Health and Medical Education and then, they were selected randomly. In selected schools, the students were selected via random sampling method. A team of trained health care professionals checked the performance of the personnel, monitored and calibrated equipment according to standard protocols. The Research Ethics Committee of the Endocrine and Metabolism Research Center (EMRC) and other relevant national regulatory organizations approved the protocol of this study. The subjects entered the survey after obtaining written consent from their parents. Quality control and quality assurance of the survey was closely supervised by the Data and Safety Monitoring Board of the project at national level.

### Physical examination

A team of trained research assistants conducted the physical examination under standard protocols, and by using calibrated instruments. Weight, height, and waist circumference (WC) were measured. Body mass index (BMI) was calculated from weight and height [BMI = weight (kg)⁄height (m^2^)]. Systolic blood pressure (SBP) and diastolic blood pressure (DBP) were recorded as the first and fifth korotkoff sounds. Blood pressure (BP) measurement were performed by using a standardized mercury sphygmomanometer on the right arm twice after a 15-min rest in a sitting position; and the mean of the two measurements was considered as the subject’s BP.

### Clinical and laboratory measurements

Blood samples were collected in the morning after a 12 hours overnight fasting. Fasting blood sugar (FBS), total cholesterol (TC), high-density lipoprotein-cholesterol (HDL-C) and triglycerides (TG) were measured enzymatically by auto-analyzers. Low-density lipoprotein-cholesterol (LDL-C) was calculated in serum samples with TG ≤ 400 mg/dl according to the Friedewald equation [13]. Serum concentration of aspartate-aminotransferase(AST) and alanine-aminotransferase(ALT) were measured by standard kits (Pars Azmoun,Tehran,Iran). Biochemical analysis was performed in the Central Provincial Laboratory that following the standards of the National Reference Laboratory, which is a collaborating center of the World Health Organization (WHO) in Tehran. Demographic information was completed by obtaining data for all officially enrolled students in the sampled classes from the school record. Family based characteristics (family history of chronic diseases [hypertension, dyslipidemia, diabetes, and obesity], parental level of education, possessing a family private car and type of home), physical activity, sedentary lifestyle, birth weight, birth order, and breast feeding duration were assessed by an interview carried out by parents or child by a trained researcher.

### Definition of terms

Abdominal obesity was defined as waist to height ratio (WHR) more than 0.5 [14]; HTN: either systolic or diastolic BP at or above the 90th percentile for age, sex and height; pre HTN:either systolic and/or diastolic BP between the 90th and 95th percentile in childhood had been designated percentile for age, sex [15]; Low HDL-C: HDL-C40(<50) mg/dl (except in boys 15–19 years old that the cut off was <45 mg/dl) ; High TG: TG100 mg/dl was taken as the 90th percentile value for age; High FBG: FBG levels of ≥100 mg/dl. High cholesterol and low- density lipoprotein cholesterol was defined according to the recommendation by the American Heart Association i.e. total cholesterol ≥200 mg/dl, LDL-C > 130 mg/dl [16]. The definition of overweight and general obesity was considered as BMI: 85th-95thand BMI >95th percentile respectively [17].

In this study, we used part of Global School-based Student Health Survey (GSHS) questionnaire from World Health Organization (WHO) for information regarding psychiatric distress. The validity and reliability of Farsi version of questionnaire was approved in Zakeri et al. [18]study. The psychiatric distress was measured by 7 questions included questions regarding worthless, angriness, anxiety, insomnia, confusion, sadness and worried problems which are presented in Table 1. If the answer to at least 4 out of the first 7 questions was C or D, or the answer to 5 or more questions was B, it counts on as a predictor of having psychiatric distress.

**Table 1:** List of questions to screen psychosocial distressaccording to Global School-based Student Health Survey (GSHS) questionnaire

**Questions**	**Response**
1. During the past 6 months how often have you felt worthless?	A. Never or rarely
2. During the past 6 months how often have you got angry too soon?	B. Once in a week
3. During the past 6 months how often have you felt anxious?	C. More than once in a week
4. During the past 6 months how often have you had a bad sleep?	D. Nearly every day
5. During the past 6 months how often have you felt dizzy or confused?	
6. During the past 12 months, have you had 2 complete weeks of sadness preventing from your routine activities?	A. Yes
	B. No
7. During the past 12 months, how often have you been so worried about something that you could not sleep at nights?	A. Never
	B. Rarely
	C. Sometimes
	D. Most of the time
	E. Always

### Statistical analysis

Data were analyzed using the STATA software version10. Quantitative variables are expressed as Means ± SD and categorical variables are expressed as percentages. Student *t*-test was used to compare mean differences between quantitative variables. Association between qualitative variables was assessed by using Pearson Chi-square test. Multiple logistic regressions(MLR) model was fitted to data to determine the association between psychosocial distressand cardio metabolic risk factors and liver enzymes after adjusting for potential confounder including age, sex, socio-economic status, parents education, birth order, family history of chronic disease, breast feeding duration, sedentary lifestyle and BMI in all abnormalities except for overweight and obesity. The results of MLR are shown as odds ratios (OR) and 95% confidence interval (CI). P-values less than 0.05 were considered as statistically significant. Design of sampling was considered in all statistical analysis.

## Results

Overall 2,812 boys and 2,781 girls participated in this cross-sectional, multi-center study. Psychosocial distress was detected in2027 (71.2%) of boys and 1759 (63.3%) of girls which was statistically significant (*P* < 0.01). Table 2 showed the baseline characteristics of participants with versus without psychiatric distress. As presented in this table, boys with psychosocial distress had higher mean of age, height and BMI than boys without psychiatric distress, but in girls only the average of age in subjects with psychosocial distress was higher compare to subjects without psychiatric distress. In both genders, the prevalence of positive family history of diabetes, obesity, dyslipidemias, high blood pressure, and osteoporosis was significantly higher in those children with psychosocial distress compared to those without it. Also, psychosocial distress was observed in 75.7% of boys and 67.9% of girls who worked more than 2 hours a day with computer.

**Table 2:** Baseline characteristics of subjects according to psychosocial distressby sex: the CASPIAN-III Study

**Variables**	**Psychosocial distressin boys**		**P-value**	**Psychosocial distressin girls**		**P-value**
	**Yes**	**No**		**Yes**	**No**	
Age (y)	14.84 ± 2.39	14.34 ± 2.53	0.001	14.85 ± 2.34	14.63 ± 2.40	0.01
BMI (kg/m^2^)^3^	19.72 ± 4.12	19.36 ± 4.10	0.04	19.24 ± 2.08	19.19 ± 4.02	0.75
Waist (cm)	68.00 ± 23.51	66.74 ± 18.65	0.18	70.24 ± 22.15	69.13 ± 12.10	0.14
Breast feeding duration (%)						
No	80.6	19.4	0.004	42.4	57.6	0.69
0-6 month	74.9	25.1		37.7	62.3	
6-12 month	77.5	22.5		36.1	63.9	
12-18 month	68.9	31.1		34.4	65.6	
18-24 month	69.4	30.6		38.2	61.8	
Family history of (%)						
Diabetes	69.9	30.1	0.20	61.8	38.2	0.007
Obesity	74.9	25.1	0.001	62.4	37.6	0.001
High blood lipids	74.2	25.8	0.001	63.9	36.1	0.001
Hypertension	73.2	26.8	0.001	63.8	36.2	0.001
Osteoporosis	73.1	26.9	0.01	65.9	34.1	0.001
Birth order (%)						
First	71.1	28.9	0.76	61.3	38.7	0.31
Second	72.2	27.8		64.4	35.6	
Third	71.6	28.4		62.0	38	
Forth or more	73.4	26.6		63.3	36.7	
Watching TV (%)						
<2 h	67.6	32.3	0.002	60.3	39.7	0.11
>2 h	73.6	26.4		63.9	36.1	
Working computer (%)						
<2 h	71.1	28.9	0.04	61.4	38.6	0.002
>2 h	75.7	24.3		67.9	32.1	
Father’s education (%)						
Illiterate	76.6	23.4	0.01	65.5	34.5	0.07
Under diploma	71.9	28.1		57.0	43.0	
University	66.5	33.5		63.1	36.9	
Mother’s education (%)						
Illiterate	76.2	23.8	0.01	67.3	32.7	0.01
Under diploma	71.4	28.6		62.4	37.6	
University	65.7	34.3		54.7	45.3	
Type of home (%)						
Personal home	70.5	29.5	0.001	37.5	62.5	0.001
Rented home	79.2	20.8		65.1	34.9	
Personal car (%)						
Yes	71	29	0.19	61.7	38.3	0.17
No	73.2	26.8		64.2	35.8	
Birth weight						
<2500gr	74	26	0.04	66.6	33.4	0.16
2500-4000gr	71.1	28.9		61.8	38.2	
>4000gr	78.2	21.8		65.1	34.9	

Mean and standard deviation of various cardio metabolic risk factors and liver enzymes according to psychosocial distress categories by sex are presented in Table 3. The mean of LDL, AST and DBP were higher and FBS and HDL were lower in boys with psychiatric distress compared to subjects without psychiatric distress. Mean of HDL and FBS in those girls with psychiatric distress was 44.94 ± 14.06 mg/dl and 87.63 ± 13.02 mg/dl, whereas in girls without psychiatric distress they were 47.65 ± 14.67 mg/dl and 88.97 ± 12.19 mg/dl, respectively which was statistically significant (P <0.05).

**Table 3:** Mean of cardiometabolic risk factors and liver enzymes according to psychosocial distresscategories by sex: the CASPIAN-III Study

	**Psychosocial distressin boys**		**P-value**^**1**^	**Psychosocial distressin girls**		**P-value**^**1**^
	** Yes**	** No**		** Yes**	** No**	
HDL-C (mg/dl)	45.88 ± 14.64	47.91 ± 13.16	0.005	44.94 ± 14.06	47.65 ± 14.67	<0.001
LDL-C (mg/dl)	87.24 ± 27.46	83.59 ± 26.60	0.01	82.92 ± 28.41	81.03 ± 25.55	0.17
TC (mg/dl)	150.86 ± 31.06	152.17 ± 32.75	0.36	144.88 ± 31.60	147.13 ± 31.84	0.10
TG (mg/dl)	94.56 ± 41.37	93.48 ± 39.61	0.55	92.70 ± 44.86	90.24 ± 43.21	0.20
SBP (mmHg)	101.61 ± 13.53	101.45 ± 13.91	0.77	104.73 ± 14.38	105.22 ± 13.20	0.39
DBP (mmHg)	65.10 ± 10.43	64.18 ± 10.57	0.03	66.82 ± 11.17	66.96 ± 10.98	0.74
FBS (mg/dl)	86.32 ± 14.28	88.56 ± 15.54	0.001	87.63 ± 13.02	88.97 ± 12.19	0.01
AST (IU/dl)	25.12 ± 14.22	23.30 ± 12.55	0.004	26.95 ± 13.40	27.36 ± 13.46	0.49
ALT(IU/dl)	17.75 ± 11.36	16.94 ± 11.43	0.14	18.73 ± 12.45	19.06 ± 10.35	0.52

HDL-C; High density lipoprotein, LDL-C; Low density lipoprotein, TC; Total cholesterol, TG; Triglyceride, SBP; Systolic blood pressure, DBP; Diastolic blood pressure, FBS; Fasting blood sugar, AST; Aspartate-aminotransferase, ALT; Alanine-aminotransferase.

^1^P-values are resulted from *T* test.

The OR and 95% CI of psychosocial distress associated with cardio metabolic risk factors and elevated liver enzymes are shown in Table 4. As presented in this table, psychosocial distresssignificantly increased the oddsof elevated LDL-C (OR = 2.36, 95%CI 1.53, 3.64) and elevated FBS (OR = 1.23, 95% CI 1.02, 1.49) and low HDL-C (OR = 1.65, 95% CI 1.41, 1.95) after adjustment for age, gender and other potential confounders. No statistically significant association was found between psychosocial distress and overweight, general obesity, abdominal obesity, high TG, high TC, pre HTN, HTN, high ALT and high AST.

**Table 4:** Odds ratios (95% CI) for cardiometabolic risk factors and liver enzymes by psychiatric distress: the CASPIAN-III Study

	**Psychosocial distress (yes/no)**		
	**OR**	**CI 95%**	**P-value**^**2**^
Overweight^1^			
Model I^3^	1.05	0.90-1.22	0.47
Model II^4^	1.02	0.88-1.19	0.71
Model III^5^	1.05	0.88-1.25	0.54
General obesity			
Model I	1.02	0.84-1.25	0.80
Model II	1.01	0.82-1.23	0.90
Model III	1.07	0.81-1.27	0.88
Abdominal obesity			
Model I	1.01	0.94-1.29	0.20
Model II	1.12	0.95-1.31	0.15
Model III	1.15	0.96-1.38	0.10
Model IV	1.26	0.99-1.60	0.06
High LDL			
Model I	2.24	1.53-3.28	0.001
Model II	2.27	1.55-3.32	0.001
Model III	2.35	1.52-3.62	0.001
Model IV	2.36	1.53-3.64	0.001
High TC			
Model I	0.82	0.63-1.06	0.13
Model II	0.81	0.62-1.04	0.11
Model III	0.85	0.64-1.13	0.27
Model IV	0.84	0.63-1.12	0.23
High TG			
Model I	1.11	0.88-1.40	0.35
Model II	1.09	0.86-1.38	0.44
Model III	1.06	0.82-1.36	0.65
Model IV	1.03	0.79-1.35	0.78
Low HDL			
Model I	1.53	1.32-1.77	0.001
Model II	1.53	1.32-1.77	0.001
Model III	1.64	1.39-1.92	0.001
Model IV	1.65	1.41-1.95	0.001
HTN			
Model I	1.08	0.14-1.38	0.53
Model II	1.08	0.84-1.40	0.51
Model III	1.28	0.96-1.72	0.09
Model IV	1.32	0.98-1.79	0.06
Pre HTN			
Model I	0.99	0.79-1.24	0.95
Model II	0.99	0.79-1.24	0.96
Model III	1.12	0.86-1.45	0.38
Model IV	1.13	0.87-1.47	0.33
High FBS			
Model I	1.25	1.49-1.05	0.01
Model II	1.20	1.42-1.01	0.04
Model III	1.21	1.47-1.02	0.03
Model IV	1.23	1.49-1.02	0.03
High ALT			
Model I	1.20	0.84-1.72	0.31
Model II	1.23	0.86-1.77	0.25
Model III	1.39	0.86-2.26	0.17
Model IV	1.31	0.81-2.14	0.26
High AST			
Model I	1.24	0.96-1.60	0.09
Model II	1.30	1.01-1.69	0.04
Model III	1.40	0.99-1.99	0.06
Model IV	1.40	0.99-2.00	0.05

1- Overweight: BMI:85th-95th; obesity: BMI > 95th; low HDL: <50 mg/dl (except in boys 15–19 years old, that cut-off was <45 mg/dl); high LDL:>110 mg/dl; high TG:> = 100 mg/dl; high TC:>200 mg/dl; high FBS:>100 mg/dl; high blood pressure:>95th adjusted by age, sex and height; ALT and AST > 40 IU/dl.

2- P-values are resulted from logistic regression.

3- Without adjusted (crude models).

4- adjusted for age and sex and living place and.other characteristics including socio-economic status, parent s education, birth order, family history of chronic disease, breast feeding duration, sedentary lifestyle.

5- Additionally adjusted for BMI in all abnormalities except for overweight and obesity.

## Discussion

In a study of 5593 students (2812 boys, 2781girls), we found that 2027 (71.2%) of boys and 1759 (63.3%) of girls had psychosocial distress. A previous investigation on 4599 girls and boys from 17 and 18-years-old adolescent in Tehran high school have found that 34.1% of girls and 23.7% of boys have psychiatric symptoms. Also, this study showed that the rate of psychiatric morbidity was higher in girl than boy students [4]. Noorbala and his colleagues performed a national investigation about mental health by using General Health Questionnaire- 24items (GHQ-24) in 35,014 individuals that 11,448 subjects of them were adolescents and young adults aged 15–24 years. These investigators demonstrated that psychosocial distresses were detected in about a fifth of population surveyed (25.9% of women and 14.9% of men). Also, women experience a high risk of psychosocial distresses than men [19]. Basirnia and his colleagues, in a systematic review reported that prevalence rates of mental health among high-school students in Iran are quite different with range of 1.9% to 58.8% [5]. According to gender distribution, girls were more likely to have psychosocial distress than boys, but the results of our study showed that boy students face to increased risk of psychosocial distress compared to girl students. Several factors may contribute to wide range prevalence of psychosocial distress in literature including temporal and geographical characteristics, differences in methods and tools for screening and diagnosis, difference in classification system, age groups of participants and small sample size [5,19].

We examined association between presences of mental illness and component of metabolic syndrome in Iranian students aged 6–18 years. After adjusting for age, gender, socio-economic status, parent’s education, birth order, family history of chronic disease, breast feeding duration, sedentary lifestyle, BMI there was direct association between mental illness and some of the parameters of metabolic syndrome including abdominal obesity, high HDL, low LDL and high FBS.

There is two-way relationship between depression and metabolic syndrome. The association between depression and component of metabolic syndrome in young and middle- age population have been shown in cross-sectional investigations [20-22], but the causality of this relationship is reminded. Data from prospective studies suggested that depression may be increased the risk of metabolic syndrome in women [23]. In the other hand, evidence from cohort studies showed that metabolic syndrome can be an important factor for the development of depression or depressive symptoms such as anxiety and anger in middle age population. This finding was observed only in women [24,25].

In a prospective cohort study of 921 participants including 538 women and 383 men in Finland, Pulkki-Raback, found that depressive symptoms in childhood were associated with increased the risk of metabolic syndrome in adulthood (OR: 1.40, CI: 1.05-1.85). Also, women with metabolic syndrome in childhood had higher level of depressive symptoms in adulthood. No association between metabolic syndrome and depression was observed in men [26]. Akbaraly and colleagues corroding to the results of Whitehall ΙΙ prospective cohort study in 5232 participants (41–61 years) have suggested that among metabolic syndrome components, the central obesity and abnormal lipids can be predictors of depressive symptoms. Hypertension had no association and high level of FBS had inverse relationship with depressive symptoms. Data from our study were consistent with these results, but in our participants there was direct association between high FBS and mental illness [27]. Data from the retrospective study in 1999–2006 on 4070 participants confirmed the association of certain factors including age, ethnic/racial status, poverty, and obesity in the diagnosis of type 2 diabetes mellitus in children. Also, it is shown the use of antidepressants and antipsychotics agents separately or in combination is associated with an increased risk of a type 2 diabetes mellitus diagnosis, weight gain and alterations in cardio metabolic indices such as dyslipidemia and hypertension in pediatric populations [28].

It is suggested that psychosocial factors can activate the HPA axis. Hyper secretion of corticotrophin-releasing hormone, adrenocortico tropic hormone and cortisol as a result of dys regulation of HPA axis can promote deposition of visceral adipose tissue [29]. Fat tissue can secrete inflammatory cytokines such as interleukin 1, 6 (IL1, IL6) and tumor necrosis factor (TNF-α). It is established that these inflammatory cytokines have been involve in insulin resistance. Also, Toschi-Dias have reported that cardiac sympathetic modulation and peripheral sympathetic activity are increased and parasympathetic modulation and heart rate variability are decreased in patients with metabolic syndrome, anxiety and mood disturbance [30]. Therefore, this autonomic dysfunction may explain the increased cardiovascular risk in patients with mood alterations.

The main limitation of this study is cross-sectional design of study which does not demonstrate the causality of association between psychosocial distresses and the cardio metabolic risk factors. Also, it is possible that residual confounder may interpret the association between psychosocial distresses and cardio metabolic risk factors. In conclusion; our findings suggest that psychosocial distresses are associated with increased risk of cardio metabolic risk factors in particular abdominal obesity, high LDL, FBS and low HDL in Iranian children and adolescents.

Strength of our study includes the large sample size and generalizability. To our knowledge, this is the first study in Iran and even in the Middle East and North Africa (MENA), to examine associations of psychosocial distresses with cardio metabolic risk factors and liver function tests in the pediatric age group.

## Competing interests

The authors declared no conflict of interests.

## Authors’ contribution

MQ, RK, HA and ET drafted the manuscript. RK, GA, MEM, BL, MRA and RH participated in study design. MQ, AR, SMA and RH participated in statistical analysis and interpretation of results. GA, MM and MC participated in data acquisition. All authors read and approved the final manuscript.

## References

[B1] Richter M Moor I van Lenthe FJ Explaining socioeconomic differences in adolescent self-rated health: the contribution of material, psychosocial and behavioral factors J Epidemiol Community Health 2012 66 8 691 697 10.1136/jech.2010.125500 21543387

[B2] De Silva MJ Huttly SR Harpham T Kenward MG Social capital and mental health: a comparative analysis of four low income countries Soc Sci Med 2007 64 1 5 20 10.1016/j.socscimed.2006.08.044 17045716

[B3] Organization WH The World Health Report: Mental Health and Physical Activity: Media centre 2011 http://www.who.int/mediacentre/multimedia/podcasts/2011/mental_health_17102011/en/index.htm

[B4] Emami H Ghazinour M Rezaeishiraz H Richter J Mental health of adolescents in Tehran Iran J Adolesc Health 2007 41 6 571 576 10.1016/j.jadohealth.2007.06.005 18023786

[B5] Basirnia A Sharifi V Mansouri N Mesgarpour B Mohammadi MR Amini H Farhoudian A Yousefi-Nooraie R Rahimi Movaghar A Prevalence of mental disorders among high-school students in Iran: a systematic review Iran J Psychiatry 2009 4 1 1 6

[B6] De Hert M Dekker JM Wood D Kahl KG Holt R Möller H-J Cardiovascular disease and diabetes in people with severe mental illness position statement from the European Psychiatric Association (EPA), supported by the European Association for the Study of Diabetes (EASD) and the European Society of Cardiology (ESC) Eur Psychiatry 2009 24 6 412 424 10.1016/j.eurpsy.2009.01.005 19682863

[B7] Ford T Goodman R Meltzer H The british child and adolescent mental health survey 1999: the prevalence of DSM-IV disorders J Am Acad Child Adolesc Psychiatry 2003 42 10 1203 1211 10.1097/00004583-200310000-00011 14560170

[B8] Mehrkash M Kelishadi R Mohammadian S Mousavinasab F Qorbani M Esmaeil M Hashemi F Asayesh H Poursafa P Shafa N Obesity and metabolic syndrome among a representative sample of Iranian adolescents Southeast Asian J Trop Med Public Health 2012 43 3 756 23077856

[B9] Mohammadi F Qorbani M Kelishadi R Baygi F Ardalan G Taslimi M Mahmoudarabi M Motlagh ME Asayesh H Larijani B Heshmat R Association of Cardio-metabolic Risk Factors and Hepatic Enzymes in a National Sample of IRANIAN Children and Adolescents: the CASPIAN-III Study J Pediatr Gastroenterol Nutr 2013 10.1097/MPG.0000000000000246 10.1097/MPG.0000000000000246 24253369

[B10] Khashayar P Heshmat R Qorbani M Esmaeil Motlagh M Aminaee T Ardalan G Farrokhi-Khajeh-Pasha Y Taslimi M Larijani B Kelishadi R Metabolic syndrome and cardiovascular risk factors in a national sample of adolescent population in the middle east and north Africa: the CASPIAN III study Int J Endocrinol 2013 Article ID 702095, 8 pages http://dx.doi.org/10.1155/2013/702095 10.1155/2013/702095 PMC3580930 23476647

[B11] Kelishadi R Abtahi S-H Qorbani M Heshmat R Esmaeil Motlagh M Taslimi M Aminaee T Ardalan G Poursafa P Moin P First National Report on Aminotransaminases’ Percentiles in Children of the Middle East and North Africa (MENA): the CASPIAN-III Study Hepat Mon 2012 12 11 e7711 12 (11) 23346152 10.5812/hepatmon.7711 PMC3546518

[B12] Kelishadi R Heshmat R Esmaeil Motlagh M Majdzadeh R Keramatian K Qorbani M Taslimi M Aminaee T Ardalan G Poursafa P Larijani B Methodology and Early Findings of the Third Survey of CASPIAN Study: a National School-based Surveillance of Students’ High Risk Behaviors IntJ Prev Med 2012 3 6 394 22783465 PMC3389436

[B13] Levy RI Estimation of the concentration of low-density lipoprotein cholesterol in plasma, without use of the preparative ultracentrifuge Clin Chem 1972 18 499 502 4337382

[B14] Knowles KM Paiva LL Sanchez SE Revilla L Lopez T Yasuda MB Yanez ND Gelaye B Williams MA Waist circumference, body mass index, and other measures of adiposity in predicting cardiovascular disease risk factors among Peruvian adults Inter J Hyper 2011 2011 Article ID 931402, 10 pages http://dx.doi.org/10.4061/2011/931402 10.4061/2011/931402 PMC3034939 21331161

[B15] Redwine KM Daniels SR Prehypertension in adolescents: risk and progression J Clin Hyper 2012 14 6 360 364 10.1111/j.1751-7176.2012.00663.x PMC3999592 22672089

[B16] Balagopal P Ferranti SD Cook S Daniels SR Gidding SS Hayman LL Mc Crindle BW Mietus-Snyder ML Steinberger J Nontraditional risk factors and biomarkers for cardiovascular disease: mechanistic, research, and clinical considerations for youth Circulation 2011 123 23 2749 2769 10.1161/CIR.0b013e31821c7c64 21555711

[B17] Flegal KM Ogden CL O'Dea JA, Eriksen MP High body mass index, overweight, and obesity in children: Definitions, terminology, and interpretation Childhood Obesity Prevention: International Research, Controversies, and Interventions 2010 Childhood obesity prevention Oxford University Press 1 10

[B18] Zakeri M Sedaghat M Motlagh ME Ashtiani RT Ardalan G BMI correlation with psychiatric problems among 10–18 years Iranian students Acta Med Iran 2012 50 3 177 184 22418986

[B19] Noorbala A Yazdi SAB Yasamy M Mohammad K Mental health survey of the adult population in Iran Br J Psychiatry 2004 184 1 70 73 10.1192/bjp.184.1.70 14702230

[B20] Kinder LS Carnethon MR Palaniappan LP King AC Fortmann SP Depression and the metabolic syndrome in young adults: findings from the third national health and nutrition examination survey Psychosom Med 2004 66 3 316 322 10.1097/01.psy.0000124755.91880.f4 15184689

[B21] Gil K Radziłłowicz P Zdrojewski T Pakalska-Korcala A Chwojnicki K Piwoński J Ignaszewska-Wyrzykowska A Załuga L Mielczarek M Landowski J Wyrzykowski B Relationship between the prevalence of depressive symptoms and metabolic syndrome. Results of the SOPKARD Project Kardiol Pol 2006 64 5 464 469 16752328

[B22] Heiskanen TH Niskanen LK Hintikka JJ Koivumaa-Honkanen HT Honkalampi KM Haatainen KM Viinamäki HT Metabolic syndrome and depression: a cross-sectional analysis J Clin Psychiatry 2006 67 9 1422 1427 10.4088/JCP.v67n0913 17017829

[B23] Raikkonen K Matthews KA Kuller LH Depressive symptoms and stressful life events predict metabolic syndrome among middle-aged women: a comparison of World Health Organization, Adult Treatment Panel III, and International Diabetes Foundation definitions Diabetes Care 2007 30 4 872 877 10.2337/dc06-1857 17392548

[B24] Raikkonen K Matthews KA Kuller LH The relationship between psychological risk attributes and the metabolic syndrome in healthy women: antecedent or consequence? Metabolism 2002 51 12 1573 1577 10.1053/meta.2002.36301 12489070

[B25] Koponen H Jokelainen J Keinanen-Kiukaanniemi S Kumpusalo E Vanhala M Metabolic syndrome predisposes to depressive symptoms: a population-based 7-year follow-up study J Clin Psychiatry 2008 69 2 178 182 10.4088/JCP.v69n0202 18232723

[B26] Pulkki-Råback L Elovainio M Kivimäki M Mattsson N Raitakari OT Puttonen S Marniemi J Viikari JSA Keltikangas-Järvinen L Depressive symptoms and the metabolic syndrome in childhood and adulthood: a prospective cohort study Health Psychol 2009 28 1 108 19210024 10.1037/a0012646 PMC3166561

[B27] Akbaraly TN Kivimäki M Brunner EJ Chandola T Marmot MG Singh-Manoux A Ferrie JE Association between metabolic syndrome and depressive symptoms in middle-aged adults results from the Whitehall II study Diabetes Care 2009 32 3 499 504 10.2337/dc08-1358 19106378 PMC2646036

[B28] Jerrell JM Tripathi A Rizvi AA McIntyre RS The risk of developing type 2 diabetes mellitus associated with psychotropic drug use in children and adolescents: a retrospective cohort analysis The Primary Care Companion to CNS Disorders 2012 14 1 PCC.11m01185 10.4088/PCC.11m01185 PMC3357575 22690363

[B29] Bjorntorp P Rosmond R The metabolic syndrome–a neuroendocrine disorder? Br J Nutr 2000 83 1 49 57 10.1017/S0007114500000088 10889792

[B30] Toschi-Dias E Trombetta IC Dias Da Silva VJ Maki-Nunes C Alves MJ Angelo LF Cepeda FX Martinez DG Negrao CE Rondon MU Symptoms of anxiety and mood disturbance alter cardiac and peripheral autonomic control in patients with metabolic syndrome Eur J ApplPhys 2012 1 9 10.1007/s00421-012-2476-8 22918560

